# Anaplastic pleomorphic xanthoastrocytoma with epithelioid morphology misdiagnosed and treated as melanoma

**DOI:** 10.1093/noajnl/vdac009

**Published:** 2022-01-25

**Authors:** Mario M Dorostkar, Dinah Konnerth, Maximilian Niyazi, Niklas Thon, Max Schlaak, Kinan Hayani, Anne Guertler

**Affiliations:** 1 Center for Neuropathology and Prion Research, Ludwig-Maximilian University (LMU), Munich, Germany; 2 Department of Radiation Oncology, University Hospital of Munich, Ludwig-Maximilian University (LMU), Munich, Germany; 3 German Cancer Consortium (DKTK), partner site Munich, Munich, Germany; 4 Department of Neurosurgery, University Hospital of Munich, Ludwig-Maximilian University (LMU), Munich, Germany; 5 Department of Dermatology and Allergy, University Hospital of Munich, Ludwig-Maximilian University (LMU), Munich, Germany

**Keywords:** astrocytoma, BRAF and MEK inhibitors, BRAF mutation, pleomorphic xanthoastrocytoma

We report on a rare case of a young patient with an intrahemispheric pleomorphic tumor, initially misdiagnosed and treated as a BRAF-mutated melanoma of unknown primary. Histologic and molecular analysis of a local recurrence 6 years later resulted in the diagnosis of an anaplastic pleomorphic xanthoastrocytoma (PXA) for both the primary and recurrent tumor. The primary tumor, assessed from stereotactic biopsies, mainly showed an epithelioid growth pattern, while the relapse had convincing astrocytic morphology. Under a treatment regime for melanoma (BRAF/MEK inhibitors) and combined stereotactic radiation, the disease was stable for 3 years.

PXA is a rare tumor, typically occurring in young adults.^1^ The histological presentation of PXA is variable, though its astrocytic origin is typically obvious. Epithelial morphology has been described in case reports of PXA, yet these cases were unequivocally of astrocytic origin.^2^ Furthermore, PXA presents with characteristic genetic alterations, with a BRAF V600E mutation and a CDKN2A inactivation most common.^3^ Lastly, a specific DNA methylation pattern is seen, which can be employed to differentiate PXA from other brain tumors.^4^ Although not a typical differential diagnosis of PXA, melanoma also metastasizes to the brain and, in rare cases, they develop intracranially.^5,6^ Around 50% of melanoma harbor a BRAF mutation, with V600E being most common.^7^

Here, we report an unusual presentation of an anaplastic PXA, which comprised mainly an epithelioid growth pattern and lacked astrocytic morphology on its initial manifestation.

## Case Presentation

**Figure 1.**
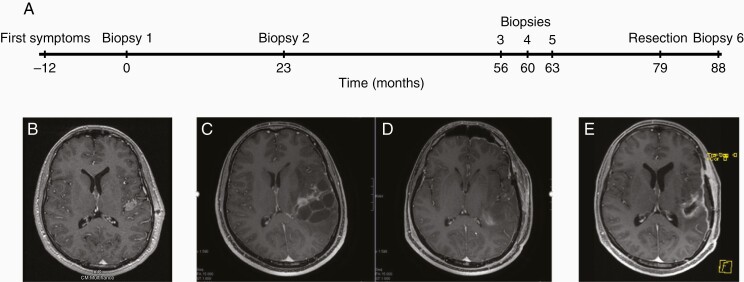
Timeline of surgical procedures and imaging results. (A) Biopsy 1 revealed an epithelioid tumor with a BRAF V600E mutation, as depicted in Figure 2 (left column). Biopsy 2 revealed a relapsing tumor with the same BRAF V600E mutation. Biopsies 3, 4, and 5 were free of tumor. The resection revealed an anaplastic PXA. Biopsy 6 was free of tumor. (B) MRI scans in March 2014, preceding biopsy 1, revealed a singular mass in the left insular cortex region (MPR sequence). (C) Pre- and (D) postoperative MRI of the recurrent tumor in September 2020 (resection; FSPGR sequence). (E) In January 2021, MPR sequence was performed 6 weeks after radiotherapy (3 months after the resection; not shown in the timeline). Abbreviations: FSPGR, fast spoiled gradient echo; MPR, multiplanar reformation; MRI, magnetic resonance imaging; PXA, pleomorphic xanthoastrocytoma.

**Figure 2.**
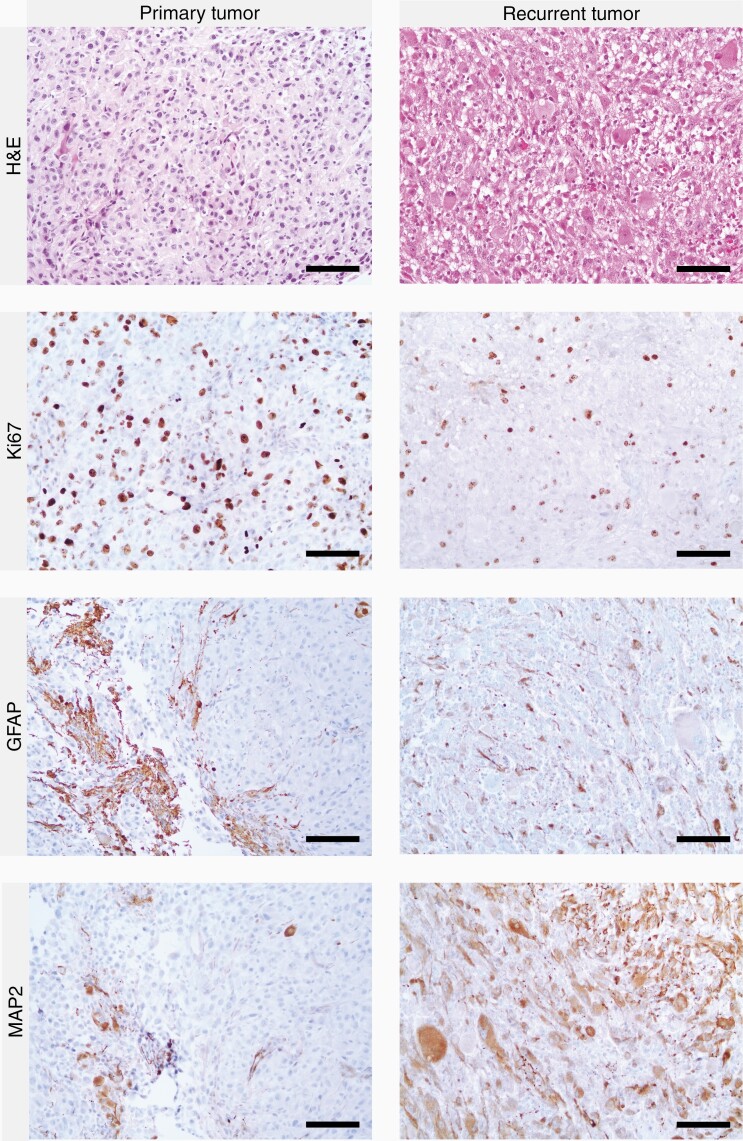
Histologic comparison of the primary tumor in March 2014 (left column) and the recurrent tumor in September 2020 (right column). The primary tumor showed pleomorphic tumor tissue without prominent astrocytic differentiation and increased mitotic/proliferative activity. GFAP and MAP2 staining was largely negative, the few positive areas were interpreted as trapped preexistent tissue. The first recurrence after 2 years showed similar characteristics (not shown). The recurrent tumor in 2020 showed higher pleomorphism and convincing astrocytic processes but lower mitotic and proliferation rates compared to the primary tumor. GFAP was expressed focally, while MAP2 expression was strong in large areas of the tumor. Scale bars = 50 µm. Abbreviations: GFAP, glial fibrillary acidic protein; MAP2, microtubule-associated protein 2.

**Figure 3.**
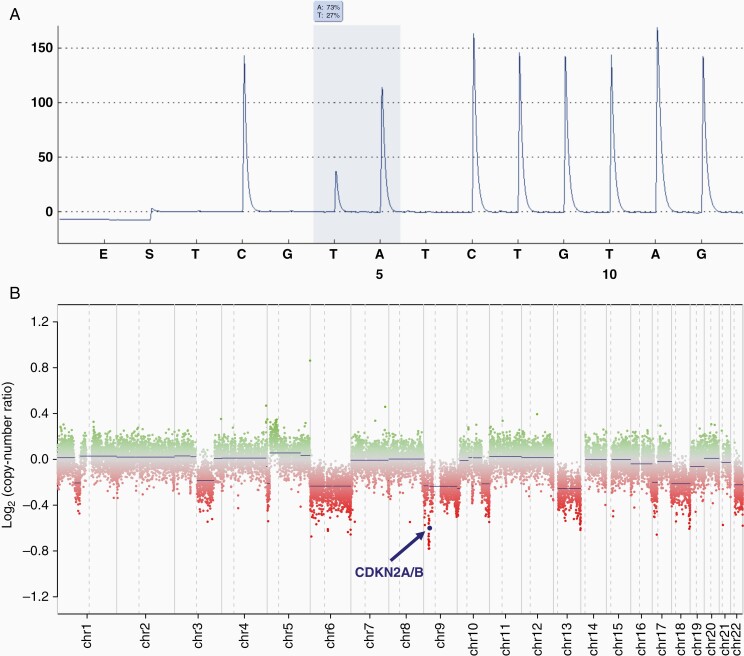
Results of genetic testing in the recurrent tumor, resected September 2020. (A) Pyrosequencing result of the BRAF V600 hotspot. The sequence shown is reverse-complimentary, thus the CAC-CTC mutation depicted corresponds to GTG-GAG in the reading frame (B) Copy-number variation plot, showing deletions of chromosomes 6, 9, 13, 18, and 22, as well as a homozygous CDKN2A/B deletion.

In March 2014, a 26-year-old Indian man (Fitzpatrick skin type IV) presented with recurrent headaches to the Department of Neurosurgery. Magnetic resonance imaging (MRI) showed a singular cystic mass in his left insular cortex region and underlying white matter (1.6 × 1.4 × 1.6 cm in diameter; Figure 1), compatible with a low-grade glioma. A stereotactic biopsy (biopsy 1) was performed, displaying a pleomorphic tumor with an increased proliferation index and mitotic rate (≥7/10 high-power fields [HPF]; Figure 2). The tumor cells lacked astrocytic processes while focal areas, immunohistochemically positive for glial fibrillary acidic protein (GFAP) and microtubule-associated protein 2 (MAP2), were interpreted as entrapped preexistent central nervous system tissue. Xanthomatous cells or extracellular eosinophilic granular bodies were not observed, although the nuclei showed prominent nucleoli. Necrosis or endothelial proliferation were not present. In silver-stained sections, only sparse reticulin fibers were seen. The tumor was faintly positive for Melan-A and negative for human melanoma black (HMB45) (not shown). Pyrosequencing revealed a BRAF V600E mutation (Figure 3A) and the results of histologic and genetic analyses were interpreted as a melanoma.

Clinical dermatological examination and additional CT scans showed no signs of further intra- or extracranial manifestations of a melanoma. In 2014, the tumor was therefore classified as a melanoma of unknown primary (MUP, pT0N0M1d(0)), clinical stage IV (American Joint Committee on Cancer [AJCC]). The patient was treated with low-activity temporary iodine-125 stereotactic brachytherapy with a reference dose (calculated to the outer rim of the tumor) of 54 Gray (Gy) (median dose rate 10.0 cGy/h).

In June 2016, MRI scans revealed a local recurrence of the cerebral mass. Stereotactic biopsy (biopsy 2) was performed and histological as well as immunohistochemical analyses presented CNS tissue infiltrated by tumor cells similar to the primary manifestation with a proliferation rate of 30%. Pyrosequencing revealed a BRAF V600E mutation. Thus, the tumor was classified as recurrent melanoma. Treatment for the recurrent tumor included stereotactic radiotherapy with a dose of 25 Gy in 5 daily fractions prescribed to the 80% isodose line, followed by combination targeted therapy with BRAF/MEK inhibitors (Dabrafenib, Trametinib). Follow-up imaging was performed every 3 months and further stereotactic biopsies were obtained in October 2018 (biopsy 3), January 2019 (biopsy 4), and May 2019 (biopsy 5), each showing reactive/necrotic alterations without vital tumor tissue, which were treated with 3 cycles of bevacizumab in 2019, according to the neuro-oncologic tumor conference.^8^ Thus, the tumor was considered as a stable disease over the course of 3 years.

In September 2020, the patient presented with increasing difficulties of speech and imaging revealed a local tumor recurrence. Radical tumor resection was performed according to the recommendation of the interdisciplinary tumor board.

## Methods

BRAF V600 hotspot pyrosequencing was performed for the initial manifestation, the first relapse, and after resection, using the Qiagen therascreen BRAF V600E RGQ PCR Kit (Qiagen, Hilden, Germany) on a Pyromark Q24 sequencer (Qiagen).

A standard diagnostic panel was performed after resection, which included pyrosequencing of the IDH R132 and IDH2 R172 hotspots using custom primers on a Pyromark Q24 sequencer as well capillary sequencing of the TERT promoter region using previously published primers^9^ on an ABI3130 sequencer (Thermo Fisher Scientific, Schwerte, Germany).

Next-generation sequencing using the Illumina Trusight Oncology 500 panel (Illumina, San Diego, CA, USA), was performed after resection. This is a combined DNA and RNA panel, evaluating 523 genes for mutations and 55 genes for fusions as well as tumor mutational burden.

Methylome analysis using the Illumina Infinium MethylationEPIC Kit and the DKFZ brain tumor methylation classifier (v11b4)^4^ was performed after resection.

The following antibodies were used for immunohistochemical stains: GFAP (M0761, DAKO, Hamburg, Germany); HMB45 (M0634, DAKO); Ki67 (M7240, DAKO); MAP2 (M4403 Merck, Ismaning, Germany); Melan-A/MART-1 (Clone A103, Thermo Fisher Scientific); Olig2 (AB9610, Millipore, Eschborn, Germany).

Informed consent was obtained from the patient for publication of this case report and accompanying data.

## Pathological Findings

Histologically, the resected tumor was unambiguously of astrocytic origin with pleomorphic tumor cells and distinct glial processes. Many tumor cells expressed GFAP and MAP2 in the cytoplasm and in processes (Figure 2). A retrospectively performed staining against Olig2 showed a heterogeneous staining pattern, with large areas negative for Olig2 and smaller areas with a variable number of tumor cell nuclei stained (Supplementary Figure 1). Retrospective staining of the primary tumor or first recurrence, unfortunately, was not feasible due to a lack of suitable tissue. Both necrosis and endothelial proliferation were now present. The proliferative index (Figure 2) and mitotic rate (4/10 HPF) were slightly lower compared to the primary manifestation. After histological assessment, an astrocytic tumor such as a radiation-induced glioblastoma, distinct from the primary tumor, was initially suspected. However, as before, genetic testing revealed a BRAF V600E mutation, while the IDH1 and IDH2 hotspots and the TERT promoter were wild-type. Methylome analysis yielded no significant match to a reference methylation class in the DKFZ brain tumor methylation classifier (v11b4).^4^ The copy-number profile showed a CDKN2A/B deletion, but no amplification of chromosome 7, loss of chromosome 10, or EGFR amplification (Figure 3B). The MGMT promoter was not methylated. Next-generation sequencing using the Illumina Trusight Oncology 500 panel revealed a LZTR1 stop mutation (NM_006767.4(LZTR1):c.628C>T (p.Arg210Ter)) in addition to the BRAF V600E mutation, but no further pathogenic alterations. The tumor mutational burden was 4/Mb with a coverage of 1.26 Mb.

With this information, the primary tumor was reclassified as an anaplastic PXA (WHO grade III) of mainly epithelioid pattern and the last tumor manifestation as its recurrence. BRAF/MEK treatment was terminated and conventionally fractionated radiotherapy with a dose of 2 Gy × 30 was initiated, analogous to treatment for high-grade glioma. In June 2021, 9 months after the resection, another biopsy (biopsy 6) was performed when a relapse was suspected on follow-up MRI scans, without vital tumor tissue.

## Discussion

In the present case, predominant epithelioid morphology in stereotactic biopsy specimens of an anaplastic PXA led to the initial diagnosis of malignant melanoma. Detection of a BRAF V600E mutation was interpreted as a confirmation of this diagnosis. Only after a bulk tumor resection of a local recurrence 6 years later, the diagnosis was revised. Notably, the initial manifestation was diagnosed before DNA methylation-based classification of brain tumors had become a routine procedure to assess difficult-to-diagnose tumors. At the time of the relapse, DNA methylation analysis of the primary tumor or first recurrence could, unfortunately, not be performed as insufficient amounts of tumor material were left. Methylation analysis of the recurrent tumor yielded no match to a reference tumor class, yet this is often the case in recurrent tumors after treatment.^4^ However, the copy-number profile showed a CDKN2A/B deletion typical for PXA and DNA/RNA panel sequencing found no alterations which could have contradicted the diagnosis.

Although most cases show concordant results in respect to histology, immunohistochemistry, molecular genetics, and clinical course, challenging cases such as the present one highlight possible limitations of diagnostic procedures in biopsy samples. For instance, due to small sample size, not all histological features may be present. In addition, the detection of typical genetic alterations in tumors (such as BRAF V600E in melanomas) does not preclude a different diagnosis, as markers are not 100% specific for a certain entity.

Notably, numerous glial or glio-neuronal tumors harbor BRAF V600E mutations.^10^ Among them, epithelioid glioblastoma is both histologically and genetically similar to aPXA and could thus be considered a differential diagnosis. The cell morphology of epithelioid glioblastoma has been described as “melanoma-like” and tumors frequently harbor BRAF V600E mutations^11^ as well as CDKN2A deletions, but typically lack xanthomatous cells or eosinophilic granular bodies.^12^ These similarities have led to speculations that the 2 entities could be related.^12,13^ Indeed, many epithelioid glioblastomas share the same DNA methylation class as PXA,^4,12^ while others cluster with conventional glioblastomas.^12^ However, epithelioid glioblastoma is reported to have a much worse prognosis than aPXA,^14,15^ which does not match the clinical course of the case described here. It should be noted, though, that the data on survival predate methylation analysis and may thus refer to such tumors that are molecularly more similar to conventional glioblastoma.

Some melanomas stain positive only for one of the markers employed in our laboratory, HMB45 and Melan-A. In this case, a very weak (and in retrospect, most likely artificial) staining with Melan-A contributed to the diagnosis of melanoma. Another commonly used marker for melanomas, MITF, could have provided valuable information in this case.

It is tempting to suggest elaborate methods such as next-generation sequencing and methylome analysis to reduce the risk of potential misdiagnoses in small samples. However, the inclusion of interdisciplinary diagnostic and clinical findings may also provide important information. The patient’s skin type confers a reduced risk to develop cutaneous melanoma and a primary melanoma which could have seeded a cerebral metastasis was not found. Furthermore, the primary radiological manifestation was not consistent with meningeal melanoma, all of which should have called the initial diagnosis into question and prompted a re-assessment.

While PXA generally has a favorable prognosis, anaplastic PXA, defined as having ≥5 mitoses/10 HPF, has a higher chance of recurrence. Alterations in the MAPK (mitogen-activated protein kinase) pathway are typical for these tumors, with mutations in the BRAF gene being most common.^16^ Because of the rarity of PXA, no formal treatment guidelines have yet been established. In WHO grade II lesions, gross total resection often produces a favorable outcome, though recurrent and WHO grade III lesions may require adjuvant therapy. However, unlike in patients with melanoma, targeted treatments with BRAF/MEK inhibitors are still the subject of current research.^17^

The presented patient experienced a progression-free interval of 3 years in recurrent anaplastic PXA upon therapy consisting of interstitial brachytherapy and adjuvant treatment with combined BRAF/MEK inhibitors. Interestingly, after this treatment regimen, the recurrent tumor presented with lower proliferation rate and mitotic count than the primary manifestation. This highlights the potential of targeted therapy in rare brain tumors, warranting further prospective randomized clinical trials such as an ongoing targeted therapy approach of Dabrafenib and Trametinib in pediatric patients with BRAF-mutated high-grade and low-grade gliomas, currently under phase II.

## Supplementary Material

vdac009_suppl_Supplementary_Figure_S1
