# Supplementary material for: Anaplastic pleomorphic xanthoastrocytoma with epithelioid morphology misdiagnosed and treated as melanoma
Source: Neurooncol Adv. 2022 Jan 25;4(1):vdac009. doi: 10.1093/noajnl/vdac009 (PMC8859830; doi:10.1093/noajnl/vdac009)

Supplementary Figure 1: Heterogeneous Olig2 staining in the resected tissue. (A-C) Selected tumor areas in the same Olig2 stained slide. Moderately dense (A) and sparse (B) staining was rare, with large areas showing no (C) labeling. Scale bars, 20 µm.


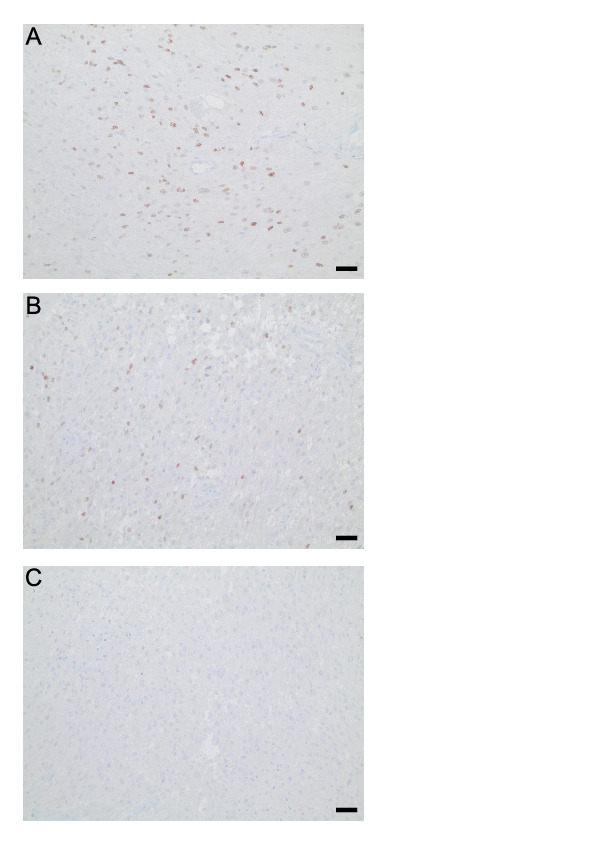

Supplement: vdac009_suppl_Supplementary_Figure_S1 [file vdac009_suppl_supplementary_figure_s1.docx]
